# Impact of molecular rapid diagnostic testing on clinical outcomes in bloodstream infections: a meta-analysis

**DOI:** 10.1186/s12879-026-13144-6

**Published:** 2026-03-24

**Authors:** Bangyao Zhou, Zhixiong Chen, Hongyi Zhu, Baihui Chen, Jing Fang, Xiaomin Jin

**Affiliations:** 1 Ruian Center for Disease Control and Prevention, Zhejiang, 32500 China; 2 Wenzhou Ouhai Center for Disease Control and Prevention, Zhejiang, 325041 China

**Keywords:** Conventional methods, Gram stain, Length of hospital stay, Mortality, Molecular rapid diagnostic testing, Intensive care unit length of stay

## Abstract

Molecular rapid diagnostic testing is considered superior to conventional microbiological methods for bloodstream infections. Molecular rapid diagnostic testing reduces time to organism identification, facilitates timely initiation of appropriate antimicrobial therapy, and improves clinical outcomes, including reduced mortality. The purpose of the study was to evaluate the impact of molecular rapid diagnostic testing on the results outcomes of bloodstream infections. We studied meta-analysis data and used a dichotomous or continuous model with fixed or random effects to get odds ratios (OR) or mean differences (MD) with 95% confidence intervals (CIs). 51 studies with a total of 14,675 subjects were selected for the study. Molecular rapid diagnostic testing was associated with significantly lower mortality (OR, 0.66; 95% CI, 0.60–0.74, *p* < 0.001), mortality with Gram-positive organisms (OR, 0.73; 95% CI, 0.59–0.90, *p* = 0.004), mortality with Gram-negative organisms (OR, 0.72; 95% CI, 0.60–0.87, *p* < 0.001), mortality with multiple organisms (OR, 0.62; 95% CI, 0.47–0.82, *p* < 0.001), length of hospital stay (MD, -3.79; 95% CI, -5.61- -1.97, *p* < 0.001), intensive care unit length of stay (MD, -2.05; 95% CI, -3.21- -0.88, *p* < 0.001), and cost (MD, -5.48; 95% CI, -9.41- -1.55, *p* < 0.001) compared to conventional methods in bloodstream infections. Molecular fast diagnostic testing for bloodstream infections significantly reduced mortality risk. Molecular fast diagnostic testing also reduced the time to successful therapy and the duration of hospitalization. Molecular fast diagnostic tests ought to be integrated into the standard of treatment for patients with bloodstream infections. However, more studies are required to validate this finding.

## Introduction

Bloostream infections are associated with high morbidity, prolonged hospitalization, and high death rate [1]. The risk of death increases when appropriate antibiotics are given late, so choosing the right antibiotic regimen early in the treatment process is very important [2]. The introduction of an adequate antibiotic therapy is influenced by the time needed to identify by culture method the bacteria and to define the antibiotic susceptibility. Bacteria identification by molecular rapid diagnostic testing is quicker than standard culture-based methods allowing a more rapid establishment of an antibiotic target therapy, that is associated to a better clinical outcome [3]. One of the five main goals of the National Action Plan for Combating Antibiotic-Resistant Bacteria is to improve molecular rapid diagnostic tests [4]. The Infectious Diseases Society of America released guidelines for their antimicrobial stewardship program, stating that to improve clinical outcomes, blood specimens should be tested quickly with help and support from the antimicrobial stewardship program [5]. The adoption of molecular rapid diagnostic testing has been limited by cost and insufficient evidence supporting its routine clinical use [6]. A meta-analysis studied the clinical benefits of molecular and phenotypic rapid diagnostic testing in bloodstream infections. However, it was limited by the fact that the studies in the meta-analysis had a short time period of follow-up [7]. This meta-analysis aimed to provide a comprehensive and current comparison of molecular rapid diagnostic testing versus conventional microbiology methods in patients with bloodstream infections, focusing on mortality, intensive care unit length of stay.

## Method

### Study design

A predefined protocol was used to conduct the meta-analysis, following established epidemiological reporting guidelines [8]. Several databases, including PubMed, Cochrane Library, Google Scholar, OVID, and Embase, were accessed in gathering and analyzing data. These datasets were applied to collect and analyze the impact of the molecular rapid diagnostic testing on the results outcomes of bloodstream infections [9]. 

### Data pooling

**Fig. 1:**
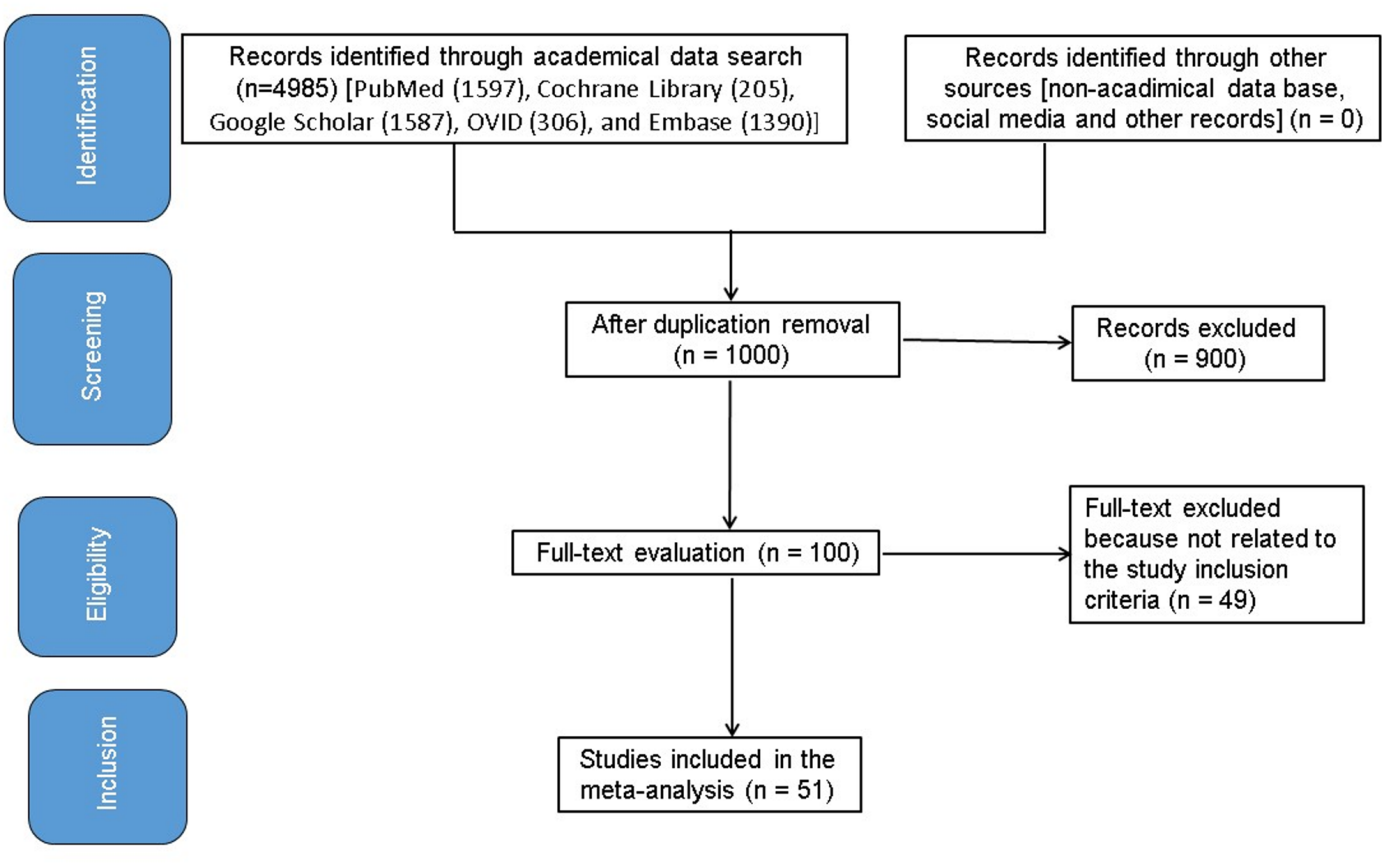
Schematic diagram of examination procedure

This meta-analysis was to assess the impact of molecular rapid diagnostic testing on the results outcomes of bloodstream infections. Language restrictions were not applied during study screening or inclusion. No limits were placed on the number of subjects in the study. Letters, reviews, and opinion articles were excluded as they do not constitute primary research suitable for meta-analysis. Figure 1 illustrates the complete inspection identification process. Gray literature and conference abstracts were evaluated.

### Eligibility of included studies

The impact of molecular rapid diagnostic testing on the result of bloodstream infections was being studied. Subclass and sensitivity analyses were applied by connecting various subtypes with intervention groups [10]. 

### Inclusion criteria and exclusion criteria

Figure 1 is a picture of the whole study. When the factors for inclusion were met, literature was added to the study:

1. Research was randomized controlled trials, retrospective, prospective, and observational.
2. Subjects with bloodstream infections were investigated as eligible subjects.
3. Interference incorporated Impact of molecular rapid diagnostic testing on clinical outcomes in bloodstream infections.
4. Study examines the impact of the molecular rapid diagnostic testing on the results outcomes of bloodstream infections.

Studies were excluded if they did not explicitly compare MRDT with conventional methods or did not report relevant clinical outcomes related to bloodstream infections.ce were excluded.

### Identification of studies

A protocol of search algorithms was established and specified by the PICOS principle [11], which states: P (population) subjects with bloodstream infections; Impact of molecular rapid diagnostic testing on clinical outcomes in bloodstream infections was I (interference); C (comparison): Comparison between molecular rapid diagnostic testing and conventional methods. O (outcome): Different results; S (study design): no limit [12]. By keywords in Table 1, we ran a thorough investigation of appropriate databases through May 2025. Assessments were run on all the studies covered in a reference management program, encompassing Author, titles, and abstracts. Also, two authors evaluate publications to detect suitable tests [13]. 

**Table 1:** Database Search Strategy for inclusion of examinations

Database	Search strategy
Google Scholar	#1 “conventional methods” OR “Gram stain” #2 “mortality” OR “molecular rapid diagnostic testing” OR “length of hospital stay” OR “intensive care unit length of stay” #3 #1 AND #2
Embase	#1 ‘conventional methods’ /exp OR ‘Gram stain’ /exp OR ‘length of hospital stay’ #2 ‘mortality’/exp OR ‘molecular rapid diagnostic testing’/exp OR ‘intensive care unit length of stay’ #3 #1 AND #2
Cochrane library	#1 (conventional methods): ti, ab, kw (Gram stain): ti, ab, kw (length of hospital stay): ti, ab, kw (Word variations have been searched) #2 (mortality): ti, ab, kw OR (molecular rapid diagnostic testing): ti, ab, kw OR(intensive care unit length of stay): ti, ab, kw (Word variations have been searched) #3 #1 AND #2
Pubmed	#1 “conventional methods“[MeSH] OR “Gram stain“[MeSH] OR “length of hospital stay” [All Fields] #2 “mortality“[MeSH Terms] OR “molecular rapid diagnostic testing“[MeSH] OR “intensive care unit length of stay“[All Fields] #3 #1 AND #2
OVID	#1 “conventional methods“[All Fields] OR “Gram stain” [All Fields] OR “length of hospital stay” [All Fields] #2 “mortality“[ All fields] OR “molecular rapid diagnostic testing“[All Fields] or “intensive care unit length of stay“[All Fields] #3 #1 AND #2

### Screening of studies

The investigation was given in a standard style, along with all of its parts. Some of the things that were used to narrow down the data were the first author’s last name, the study’s date, the country where it took place, the gender of the subjects who took part, the total number of subjects, their clinical characteristics, demographic information, and the qualitative and quantitative evaluation methods that were used [14]. The authors studied the possibility of bias in the selected studies and the quality of the methods used in papers that were chosen for further research. Two authors studied the methods used for each test without bias [15]. 

### Reporting bias assessment

We employed the Egger regression test and funnel plots, which illustrate the logarithm of odds ratios against their standard errors, to assess study bias both mathematically and intuitively. The presence of investigation bias was determined by *p* ≥ 0.05 [11]. Two authors evaluated the selected papers’ methods independently in order to assess the possibility of bias in each study. Procedural quality was assessed using the “risk of bias instrument” from the Cochrane Handbook for Systematic Reviews of Interventions, Version 5.1.0. After each study was classified using the assessment criteria, it was given one of the following bias risks: The research was categorized as having a medium bias risk if one or more quality requirements weren’t met, and as having a low bias risk if all requirements were met. The research was deemed to have a significant bias risk if multiple quality standards were either fully or partially satisfied.

### Statistical analysis

We used a dichotomous or continuous model with fixed or random effects to measure the odds ratio (OR) or mean difference (MD) with a 95% confidence interval (CI) [11]. The I^2^ index that has been calculated is given as a number and has a range of 0 to 100. Higher I² values indicate greater heterogeneity among studies, whereas lower values suggest less heterogeneity. If I^2^ was 50% or more, a random effect was picked. If it was not, a fixed effect was picked [16]. As part of subcategory analysis, the results of the study were grouped. Egger’s tests were used for quantitative analysis to measure bias. Bias was thought to exist if *p* > 0.05 [17, 18]. A two-tailed method was used to figure out the p-values. Graphs and statistical studies were made with Review Manager 5.4 (The Nordic Cochrane Center, the Cochrane Collaboration, Copenhagen, Denmark).

## Results

After examining 4985 relevant publications, 51 studies that were published between 2006 and 2024 satisfied the necessities and were covered in this study [19–69]. Table 2 summarizes findings of these studies. A total of 14,675 patients were included in the analysis.

**Table 2:** Characteristics of studies

Study	Country	Total	Molecular rapid diagnostic testing	Conventional methods
Forrest a, 2006 [19]	USA	148	72	76
Forrest b, 2006 [20]	USA	203	119	84
Forrest, 2008 [21]	USA	224	95	129
Ly, 2008 [22]	USA	202	101	101
Neuberger, 2008 [23]	Palestine	84	42	42
Bauer, 2010 [24]	USA	156	82	74
Holtzman, 2011 [25]	USA	199	99	100
Perez, 2012 [26]	USA	219	107	112
Frye Abigail, 2012 [27]	USA	244	110	134
Heil, 2012 [28]	USA	82	21	61
Huang, 2013 [29]	USA	501	245	256
Sango, 2013 [30]	USA	74	28	46
Wang, 2013 [31]	Canada	86	48	38
Maslonka, 2014 [32]	USA	110	55	55
Nagel Jerod, 2014 [33]	USA	246	117	129
Beuving, 2015 [34]	Netherlands	223	114	109
Box, 2015 [35]	USA	167	64	103
Macvane, 2015 [36]	USA	113	63	50
Sothoron, 2015 [37]	USA	126	67	59
Felsenstein, 2016 [38]	USA	383	189	194
Malcolmson, 2016 [39]	Canada	221	121	100
Lockwood, 2016 [40]	USA	390	241	149
MacVane, 2016 [41]	USA	68	23	45
Pardo, 2016 [42]	USA	336	84	252
Walker, 2016 [43]	USA	195	97	98
Patel, 2017 [44]	USA	480	233	247
Rivard, 2017 [45]	USA	877	421	456
Avdic, 2017 [46]	USA	264	137	127
Beganovic, 2017 [47]	USA	252	126	126
Eby, 2017 [48]	USA	226	120	106
Jeon, 2018 [49]	Korea	556	254	302
Box, 2019 [50]	USA	1051	539	512
Verroken, 2019 [51]	Belgium	287	139	148
Banerjee, 2020 [52]	USA	448	222	226
Hogan Catherine, 2020 [53]	USA	671	335	336
Claeys, 2020 [54]	USA	832	595	237
Sheth, 2020 [55]	USA	173	89	84
Mahrous, 2020 [56]	Saudi Arabia	312	148	164
AlQahtani, 2021 [57]	Saudi Arabia	39	14	25
Wade-Cummings, 2021 [58]	Canada	197	98	99
Kim, 2021 [59]	Korea	116	56	60
Anton-Vazquez, 2021 [60]	UK	191	93	98
Valentin, 2021 [61]	Austria	426	111	315
Berinson, 2021 [62]	Germany	105	51	54
Christensen, 2022 [63]	USA	205	108	97
McCarthy, 2022 [64]	USA	199	101	98
Al Sidairi, 2023 [65]	Canada	56	29	27
Weng, 2023 [66]	China	1004	519	485
Bae, 2023 [67]	Korea	358	144	214
Yetukuri, 2024 [68]	USA	200	100	100
Keri, 2024 [69]	India	150	100	50
	Total	14,675	7286	7389

**Fig. 2:**
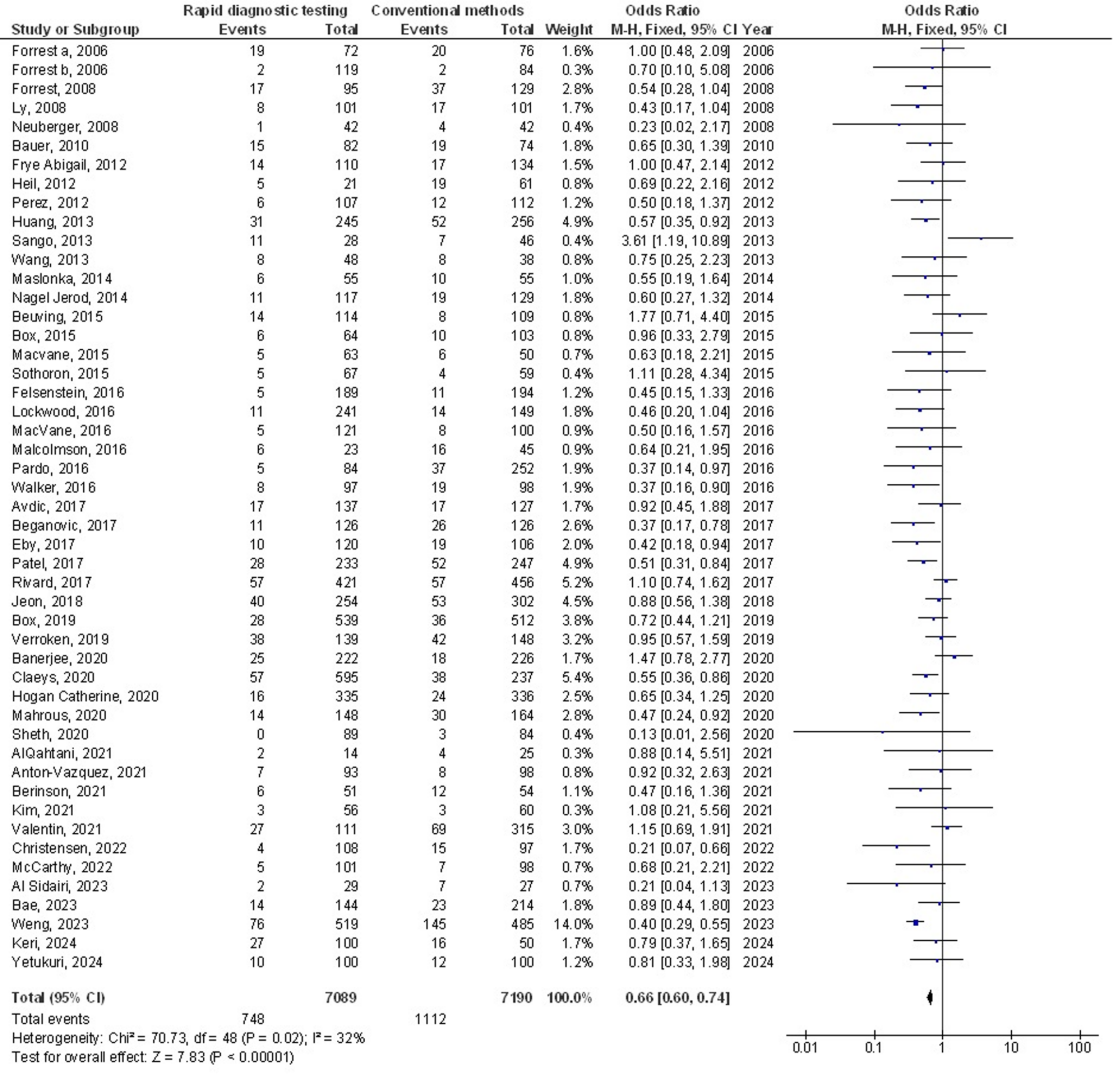
Effects of the forest plot of molecular rapid diagnostic testing compared to control on mortality in bloodstream infections

**Fig. 3:**
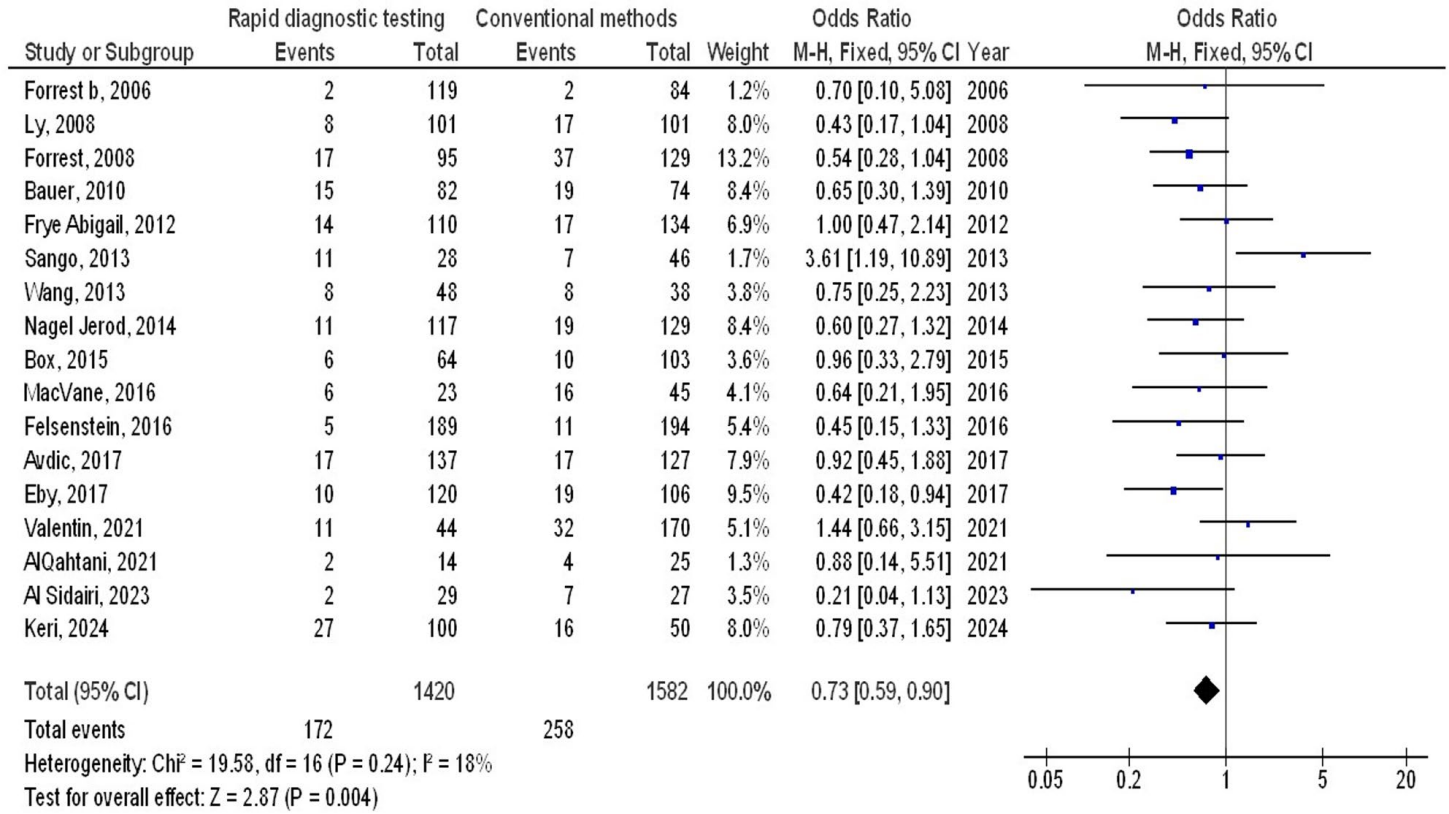
Effects of the forest plot of molecular rapid diagnostic testing compared to control on mortality with Gram-positive organisms in bloodstream infections

**Fig. 4:**
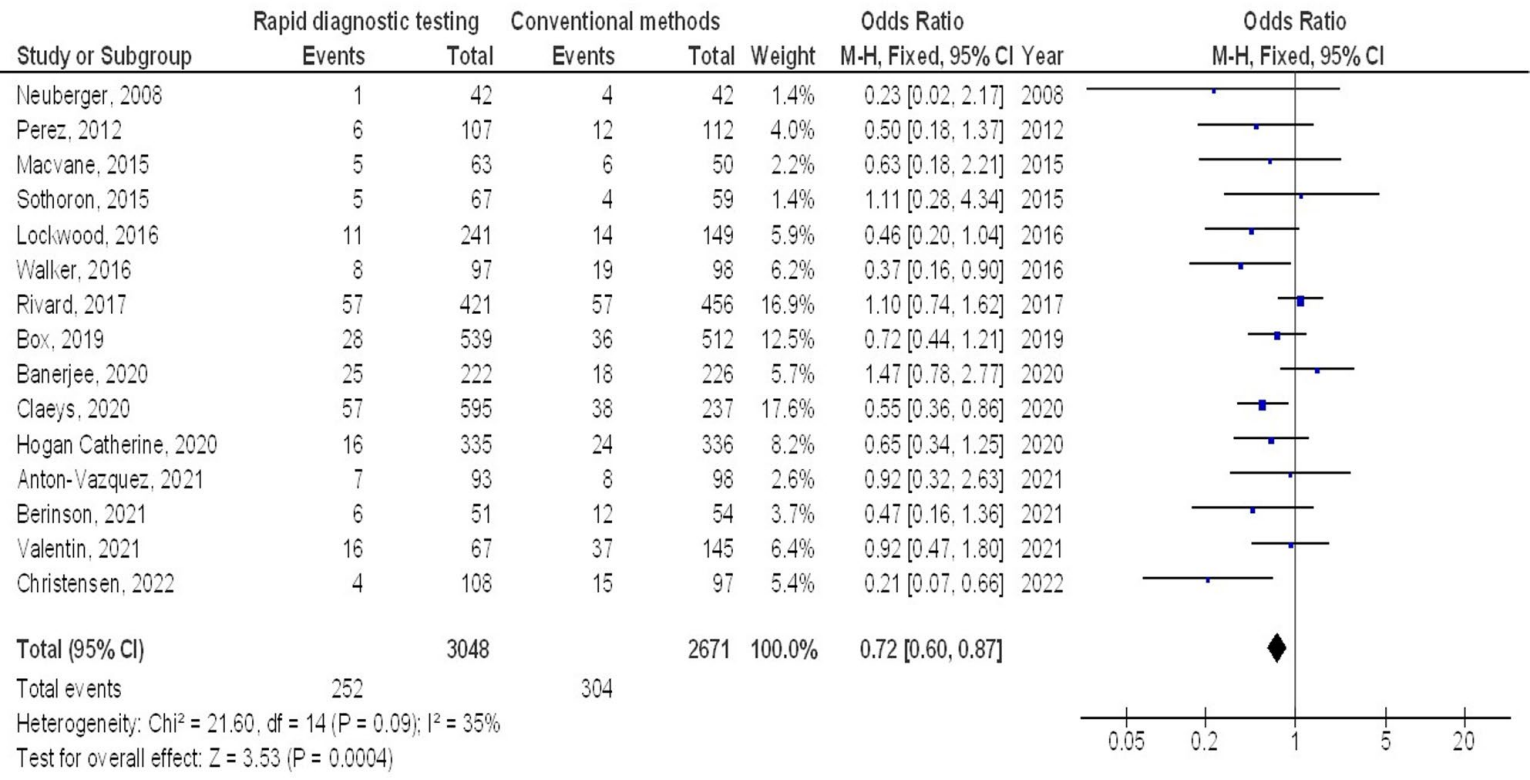
Effects of the forest plot of molecular rapid diagnostic testing compared to control on mortality with Gram-negative organisms in bloodstream infections

**Fig. 5:**
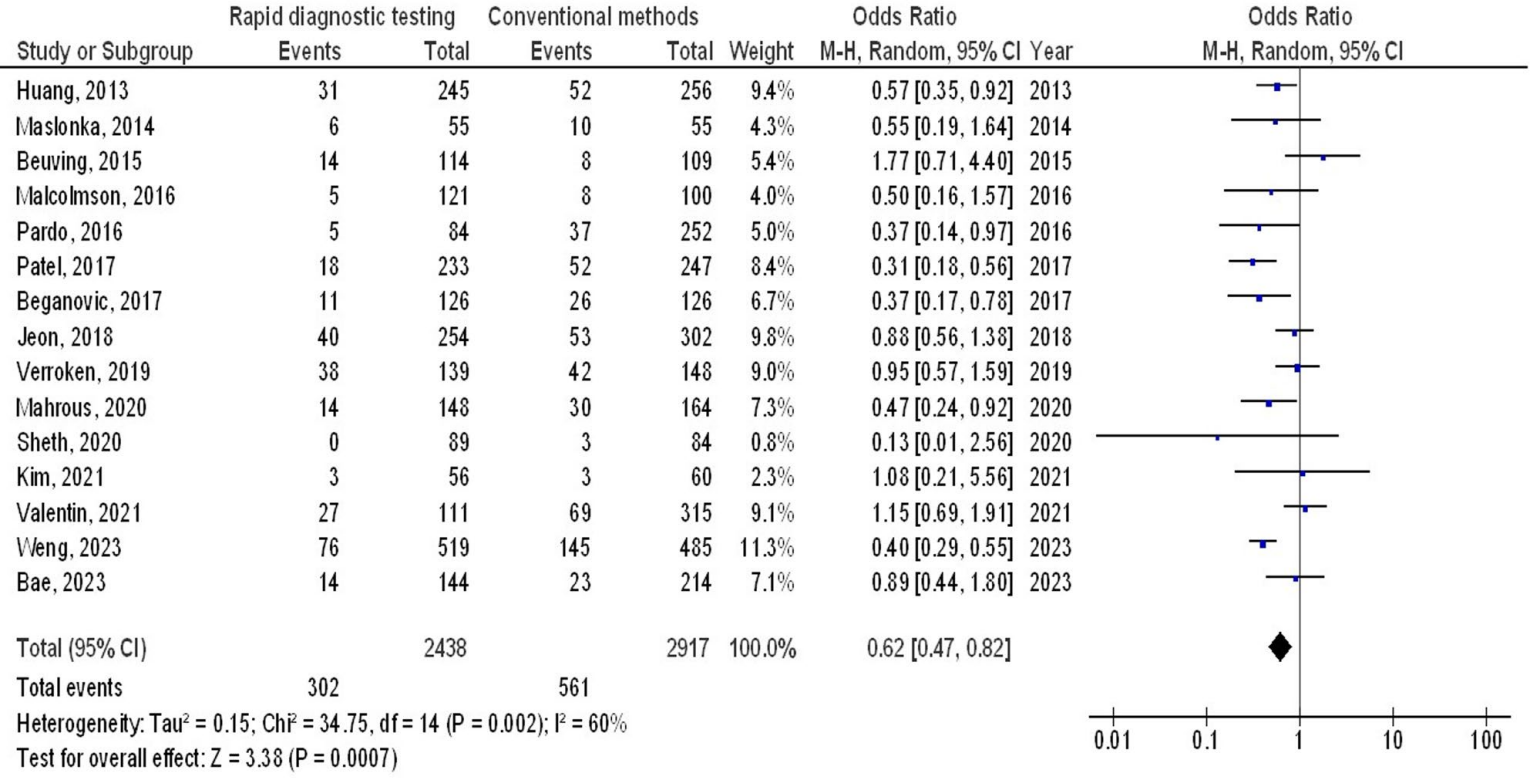
Effects of the forest plot of molecular rapid diagnostic testing compared to control on mortality with multiple organisms in bloodstream infections

**Fig. 6:**
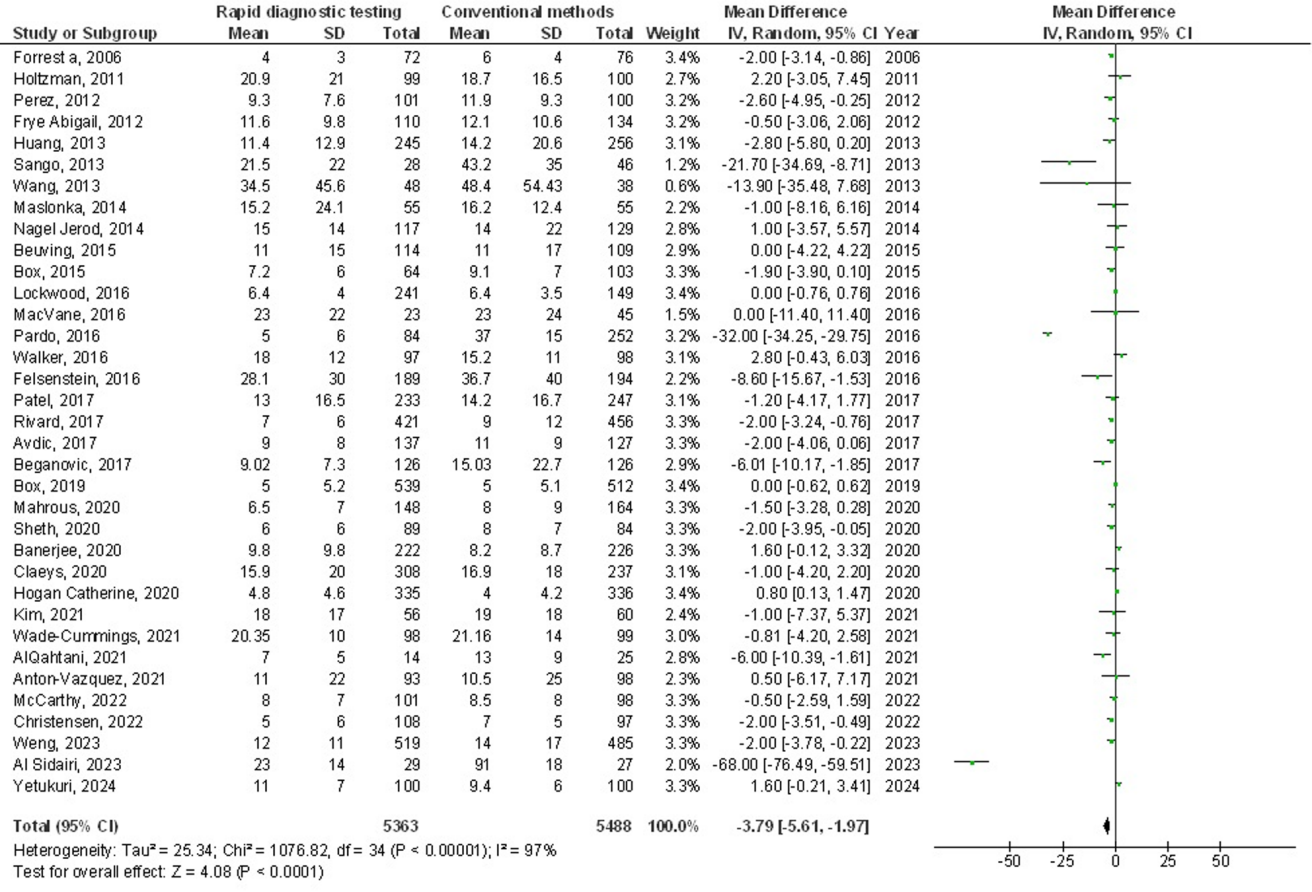
Effects of the forest plot of molecular rapid diagnostic testing compared to control on the length of hospital stay in bloodstream infections

**Fig. 7:**
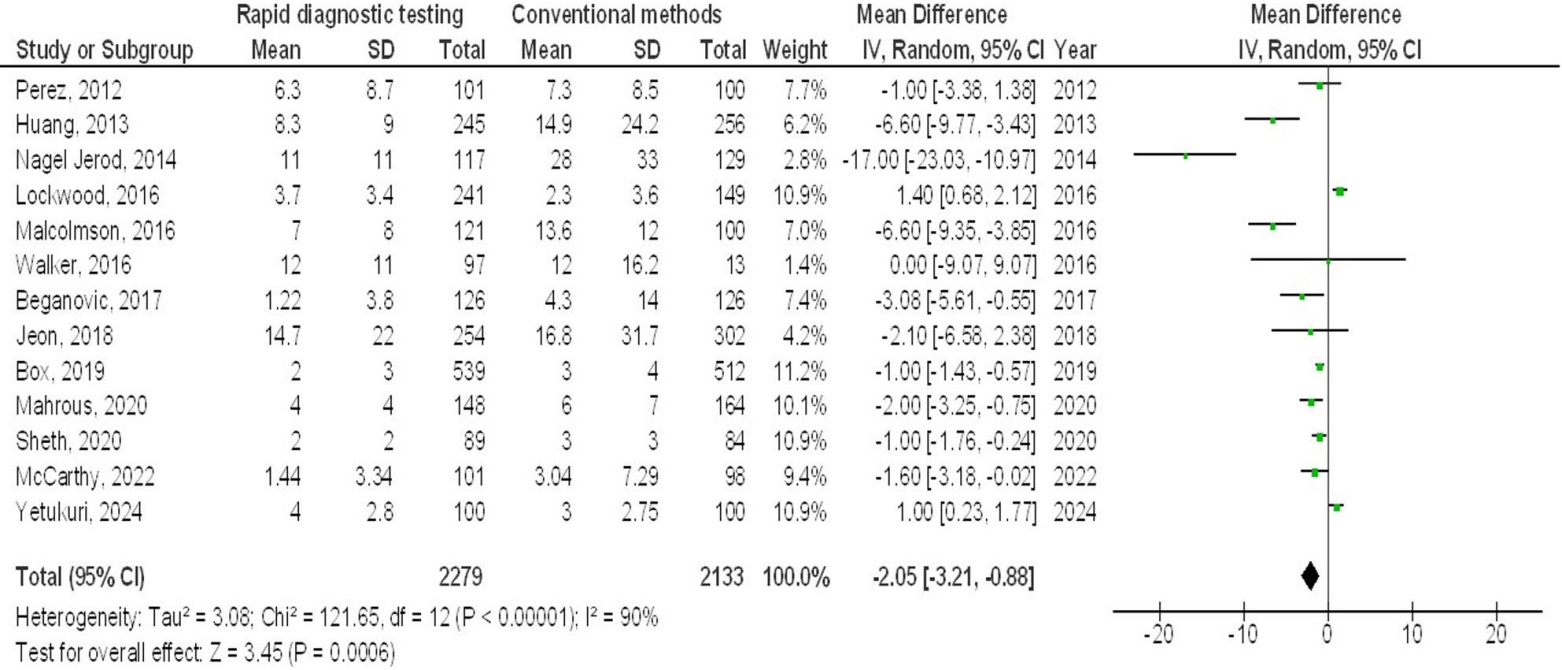
Effects of the forest plot of molecular rapid diagnostic testing compared to control on intensive care unit length of stay in bloodstream infections

**Fig. 8:**
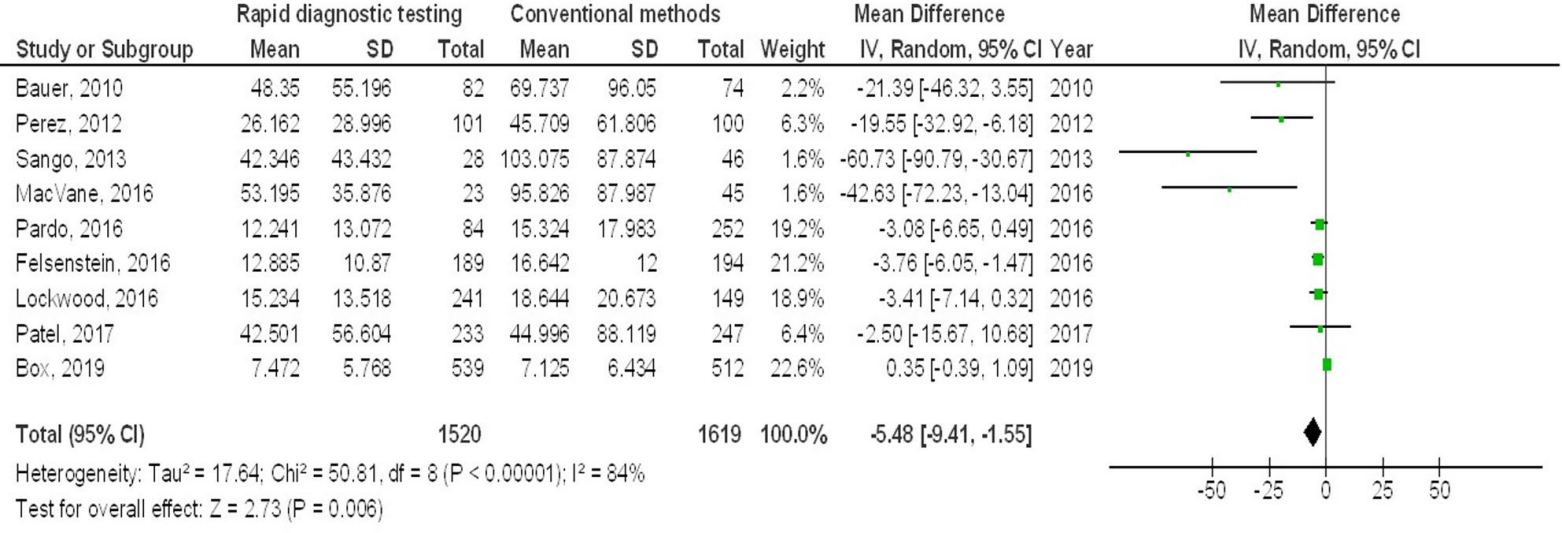
Effects of the forest plot of molecular rapid diagnostic testing compared to control on cost in bloodstream infections

Molecular rapid diagnostic testing was associated with significantly lower mortality (OR, 0.66; 95% CI, 0.60–0.74, *p* < 0.001) with low heterogeneity (I^2^ = 32%), mortality with Gram-positive organisms (OR, 0.73; 95% CI, 0.59–0.90, *p* = 0.004) with no heterogeneity (I^2^ = 18%), mortality with Gram-negative organisms (OR, 0.72; 95% CI, 0.60–0.87, *p* < 0.001) with low heterogeneity (I^2^ = 35%), mortality with multiple organisms (OR, 0.62; 95% CI, 0.47–0.82, *p* < 0.001) with moderate heterogeneity (I^2^ = 60%), length of hospital stay (MD, -3.79; 95% CI, -5.61- -1.97, *p* < 0.001) with high heterogeneity (I^2^ = 97%), intensive care unit length of stay (MD, -2.05; 95% CI, -3.21- -0.88, *p* < 0.001) with high heterogeneity (I^2^ = 90%), and cost (MD, -5.48; 95% CI, -9.41- -1.55, *p* < 0.001) with high heterogeneity (I^2^ = 84%) compared to conventional methods in bloodstream infections, as shown in Figs. 2, 3, 4, 5, 6, 7 and 8.

The quantitative Egger regression test and visual inspection of funnel plots revealed no significant publication bias (*p* = 0.85). It was exposed that the mainstream of selected studies had poor practical quality and were prejudiced in their selective reporting.

## Discussion

For the current meta-analysis, 51 studies with A total of 14,675 patients were included in the analysis [19–69]. The use of imprecise or incomplete data from previous studies may have exacerbated potential bias., mortality with Gram-positive organisms, mortality with Gram-negative organisms, mortality with multiple organisms, length of hospital stay, intensive care unit length of stay, and cost compared to conventional methods in bloodstream infections. Nevertheless, further studies are needed to confirm these results and assess generalizability [70, 71]. 

It is not known how widely molecular rapid diagnostic testing for bloodstream infections is used in acute care facilities in the USA. However, the National Action Plan for Combating Antibiotic-Resistant Bacteria has called for its use to find drug-resistant organisms and improve patient safety [72]. Molecular rapid diagnostic testing has been shown to improve clinical outcomes in a number of observational studies [73]. However, a randomized control trial suggests that these technologies do not have much of an effect [73]. In this study, however, normal blood culture processing included matrix-assisted laser desorption/ionization-time of flight analysis; hence, molecular rapid diagnostic testing was used in both groups that were compared.

We believe our results back up the suggestion from the Infectious Diseases Society of America’s antimicrobial stewardship program guideline to use molecular rapid diagnostic testing with help from the antimicrobial stewardship program for bloodstream infections [74]. The Molecular rapid diagnostic testing should be considered a standard component of care for patients with bloodstream infections, particularly when integrated with antimicrobial stewardship. Molecular rapid diagnostic testing was linked to a significantly lower risk of death compared to traditional microbiological methods. This was true for studies that included gram-negative organisms, gram-positive organisms, and multiple types of infections. It was also shown that molecular rapid diagnostic testing might help shorten the time it takes to start effective therapy for vancomycin-resistant enterococci [75]. Our findings show that molecular rapid diagnostic testing can be very helpful in finding vancomycin-resistant enterococci bloodstream infections. This can cut the time it takes to start effective treatment by more than 24 h. Also, for all studies of vancomycin-resistant enterococci that we studied, the average time to effective treatment was between 43.7 and 50.2 h. Therefore, molecular rapid diagnostic testing may be particularly beneficial for patients with vancomycin-resistant enterococci bacteremia and could contribute to reduced mortality in this population. Finally, molecular rapid diagnostic testing was associated with reductions in hospital length of stay, intensive care unit length of stay, and overall cost. The shorter stays we observed have big effects on costs because they save money every day that the subject does not have to stay in the hospital. When the prices of molecular rapid diagnostic testing for bloodstream infections were taken into account, a study that evaluated the economic effects of the test found that it saved about $30,000 per 100 patients [42]. However, the reported shorter stays are probably only true in big hospitals and medical centers, since only two of the studies were done in community hospitals. Also, length of stay, and intensive care unit length of stay did not go down significantly in the two studies that took into account confounders [34, 42], but the results are hard to draw conclusions from because of the large number of variables and small sample size. There are some problems with our meta-analysis. Even though our results may only apply to University medical centers when it comes to clinical outcomes, it’s important to note that two studies from community hospitals were included [35, 40]. In one of these studies, there was an antimicrobial stewardship program, but the bloodstream infections were treated by pharmacists who were not skilled in infectious diseases [40]. Best practices in this area would become clearer if more studies were done in neighborhood hospitals to show how things turned out. Researchers should follow the instructions given on how to record and share these results when using molecular rapid diagnostic testing for bloodstream infections in the future [76]. We also treated all interventions the same when it came to the type of technology used. This is because different labs do things differently, like mixing up tests or using matrix-assisted laser desorption/ionization-time of flight analysis on samples taken directly from blood culture bottles with nutritional broth or from solid agar that was left to sit overnight. Different ways were used to determine the results of molecular rapid diagnostic testing, which could affect the patients’ status in the long run. In the future, researchers may study these differences and how they affect clinical results, but our study shows that molecular rapid diagnostic testing as a whole makes things better for people with bloodstream infections. We recommend that molecular rapid diagnostic testing implementation be accompanied by structured protocols to ensure accurate interpretation and real-time reporting of results, reported in real time, and used to help choose the best treatment. If there are tests available 24 h a day, seven days a week, and the provider is notified right away, along with guidance from an antimicrobial stewardship program team, treatment can be started, stepped up, or stepped down more quickly.

Further research is needed in community hospital settings to evaluate the combined impact of molecular rapid diagnostic testing and antimicrobial stewardship programs on clinical outcomes and to establish best practices. Based on the results, molecular rapid diagnostic testing should be thought of as normal care for people who have infections in their bloodstreams.

The Cochrane risk of bias assessment tool was used to check the quality of the studies that were included. The quality of most of the studies that were included was high to moderate across all sub-domains. Because of this, the total risk of bias in the studies that were selected is low to moderate. Multicenter randomized controlled trials that last longer are needed to find out how well molecular rapid diagnostic testing reduces medical mortality.

### Strengths and limitations

The strengths of our meta-analysis were the inclusion of a large number of studies, and most of the comparisons include many studies. A potential limitation is selection bias, which may have arisen if relevant studies were inadvertently excluded. Nevertheless, all excluded work did not meet the necessary criteria to be included in the study. Also, bias could be due to different ways used to determine the results of molecular rapid diagnostic testing, which could affect the patients’ status in the long run. Still, data were needed to determine whether influences, e.g., ethnicity, age, and gender, influenced the consequence. The study aimed to define the impact of molecular rapid diagnostic testing on the results outcomes of bloodstream infections. The use of imprecise or incomplete data from previous studies may have exacerbated potential bias. A person’s age, gender, ethnicity, and nutritional state were the main variables that most likely contributed to discrimination. Values may unintentionally be modified as a result of unreported investigations and inadequate data.

## Conclusions

The use of imprecise or incomplete data from previous studies may have exacerbated potential bias., mortality with Gram-positive organisms, mortality with Gram-negative organisms, mortality with multiple organisms, length of hospital stay, intensive care unit length of stay, and cost compared to conventional methods in bloodstream infections. However, more studies are required to validate this finding. In the future, researchers may study the effect of differences in molecular rapid diagnostic testing and how they affect clinical results, but our study shows that molecular rapid diagnostic testing as a whole makes things better for people with bloodstream infections.

## Data Availability

The datasets analyzed during the current study are available from the corresponding author on reasonable request.
